# Recombinant Marek’s Disease Virus as a Vector-Based Vaccine against Avian Leukosis Virus Subgroup J in Chicken

**DOI:** 10.3390/v8110301

**Published:** 2016-11-04

**Authors:** Yongzhen Liu, Kai Li, Yulong Gao, Li Gao, Li Zhong, Yao Zhang, Changjun Liu, Yanping Zhang, Xiaomei Wang

**Affiliations:** Avian Immunosuppressive Diseases Division, State Key Laboratory of Veterinary Biotechnology, Harbin Veterinary Research Institute, Chinese Academy of Agricultural Sciences, Harbin 150069, China; yongzhenhvri@163.com (Y.L.); likai01@caas.cn (K.L.); ylg@hvri.ac.cn (Y.G.); gaoli0820@163.com (L.G.); lizimd@163.com (L.Z.); zhangyao502@126.com (Y.Z.); liucj93711@hvri.ac.cn (C.L.); zhangyanping03@caas.cn (Y.Z.)

**Keywords:** avian leukosis virus subgroup J, recombinant Marek’s disease virus, vaccine, chicken

## Abstract

Avian leukosis virus subgroup J (ALV-J) is an immunosuppressive virus that causes considerable economic losses to the chicken industry in China. However, there is currently no effective vaccine to prevent ALV-J infection. In order to reduce the losses caused by ALV-J, we constructed two effective ALV-J vaccines by inserting the ALV-J (strain JL093-1) *env* or *gag*+*env* genes into the *US2* gene of the Marek’s disease herpesviruses (MDV) by transfection of overlapping fosmid DNAs, creating two recombinant MDVs, rMDV/ALV-gag+env and rMDV/ALV-env. Analysis of cultured chicken embryo fibroblasts infected with the rMDVs revealed that Env and Gag were successfully expressed and that there was no difference in growth kinetics in cells infected with rMDVs compared with that of cells infected with the parent MDV. Chickens vaccinated with either rMDV revealed that positive serum antibodies were induced. Both rMDVs also effectively reduced the rate of positive viremia in chicken flocks challenged with ALV-J. The protective effect provided by rMDV/ALV-env inoculation was slightly stronger than that provided by rMDV/ALV-gag+env. This represents the first study where a potential rMDV vaccine, expressing ALV-J antigenic genes, has been shown to be effective in the prevention of ALV-J. Our study also opens new avenues for the control of MDV and ALV-J co-infection.

## 1. Introduction

Since avian leukosis virus subgroup J (ALV-J) was first described, it has spread, leading to serious economic losses in poultry production [1]. Both broilers and layers can be infected, inducing the formation of various types of tumors including hemangiomas and myelocytomas [2,3]. ALV-J-induced diseases are particularly seen among layer chickens and local chickens in China [4], and infection patterns in the country differ from those observed in Europe, where no layer cases have been found. This finding indicates that the host range of ALV-J has gradually expanded in China. Controlling and eradicating ALV-J in China is challenging because of the widespread distribution of this virus in layer flocks as well as the lack of organization in the poultry industry. Furthermore, owing to the various strains and large population of local chickens and to the non-standard and high-density farming techniques used in China, achieving a very low ALV-J infection rate in local chickens is likely to be a difficult and costly process, which will require purification of the population [5,6]. In order to control and prevent ALV-J epidemics and to protect the local chicken species in China, control methods and measures should be utilized to reduce the rate of ALV-J infection. Vaccine-based prevention is an effective control method [7,8,9]. However, there is currently no effective vaccine to prevent ALV-J infection.

Marek’s disease is a multifaceted disease characterized by the induction of rapid and extensive malignant T-cell lymphoma. It is caused by oncogenic (serotype 1) strains of Marek’s disease herpesvirus (MDV) [10,11]. Marek’s disease can be effectively prevented by vaccination with attenuated MDV or herpesvirus of turkey (HVT) [12,13]. As with most herpesviruses, MDV exhibits several characteristics that make it a good vector for recombinant vaccines [14,15,16]. MDV has a large genome with several regions that are nonessential for viral replication, allowing the expression of foreign antigen genes from other pathogens [15,16]. Furthermore, MDV establishes a persistent infection in the lymphoid tissues and can induce long-term immune responses [11]. An MDV-vectored live vaccine targeted against ALV-J could help to control ALV-J infection by preventing its spread in chickens. Therefore, the main goal of this study was to evaluate MDV as a vector for the delivery of a vaccine capable of protecting chickens against ALV-J.

In recent years, many researchers in China have tried to find an effective vaccine to help the ALV purification process. However, the research showed that the inactivated vaccine cannot produce enough antibodies to protect against ALV-J infection, and the attenuated vaccine poses high risk for reactivation and infection [8]. A number of studies have suggested that a subunit vaccine containing gp85 protein plus CpG-oligodeoxynucleotides (ODN) adjuvant can induce breeder hens not only to produce higher amounts of serum antibody against ALV-J, but also to produce protective maternal antibody in hatched chickens’ sera [7,8,9]. Therefore, the genetic engineering vaccine could be a good choice for the prevention of ALV-J infection. In this study, we generated two effective MDV-based ALV-J vaccines by expressing the ALV-J *env* or *env*+*gag* antigens in a serotype 1 MDV vaccine strain. Our results demonstrate that these MDV-vectored live vaccines induce an immune response and provide protection against ALV-J infection.

## 2. Materials and Methods

### 2.1. Viruses and Cells

The avirulent MDV1 814 strain [17] and recombinant viruses were propagated in monolayers of chicken embryo fibroblasts (CEFs) prepared from 10-day-old specific-pathogen-free (SPF) embryos. ALV-J strain JL093-1 (GenBank accession number: JN624878.1), isolated in Jilin Province in China in 2009 from a commercial layer chicken with hemangiomas, was stored at −70 °C and propagated in the DF1 cell line [4].

### 2.2. Construction of Fosmids

We constructed two cassettes: one for the expression of the ALV-J *env* gene and one for expression of the ALV-J *gag* and *env* genes with an internal ribosome entry site [18] between the two genes (*gag*-IRES-*env*). First, the *env* and *gag* genes were amplified from JL093-1 using polymerase chain reaction (PCR) with a pair of primers specific for *env* genes (5′-TTTCCCGGGGCCACCATGGAAGCCGTCATAAAGGCATTTCTGACTGGGCACCC and 5′-TTTCTCGAGCTACAGTTGCTCCCTAATTCTA) and a pair specific for *gag* genes (5′-TTTGAGCTCGCCACCATGGAAGCCGTCATAAAGGTGA and 5′-TTTATCGATCTATAAATTTGTCAAGCGGAGC). The *env* and *gag* genes were then introduced into the pCAGGS plasmid to form the *env* gene-expressing cassette (CAG-*env*) and the *gag-*IRES*-env* gene-expressing cassette (CAG-*gag*-IRES-*env*), respectively. A woodchuck hepatitis virus posttranscriptional regulatory element (WPRE) was then inserted between the antigen gene and the poly(A) to enhance the expression of the foreign gene [19]. These cassettes included the cytomegalovirus (CMV) enhancer, chicken β-actin promoter, *env* open reading frame (ORF) or *gag*-IRES-*env* ORF, WPRE, and Simian virus 40 (SV40) poly(A) signal. The CAGW-env and CAGW-gag-IRES-env cassettes were then ligated into a versatile entry vector, pENTR-mcs, derived from plasmid pENTR-gus (Invitrogen, Carlsbad, NM, USA), containing a multiple cloning sequence flanked by attL1 and attL2 sequences. The resulting entry plasmids were designated pENTR-CAG-env and pENTR-CAG-gag-IRES-env.

**Figure 1:**
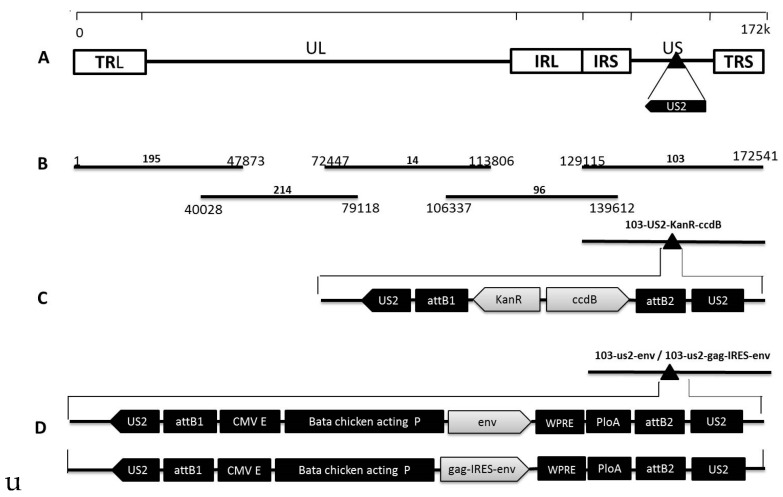
Construction the recombinant fosmid with *env* and *gag-IRES-env* genes inserted at the *US2* site in the Marek’s disease herpesviruses (MDV) genome. (**A**) the genomic structure of the MDV 814 strain; (**B**) the five fosmid DNAs used for MDV regeneration. Numbers show the fosmid names and the location of each fosmid fragment in the genome of the 814 strain; (**C**) the schematic diagrams of the mutant fosmid inserted with the Kan-ccdB dual selection markers flanked by the attR1 and attR2 sequences within the *US2* gene; and (**D**) the schematic diagrams of recombinant fosmid inserted with the CAG-env or CAG-gag-IRES-env expression cassette within the US2 gene.

Five fosmids (195, 214, 14, 96, and 103) (Figure 1B) containing sequences spanning the entire genome of MDV1 strain 814 (Figure 1A) were constructed in our preliminary studies for the rescue of the parental virus. To facilitate the insertion of foreign genes into the MDV genome, a dual selection cassette encoding the KanR and ccdB markers and flanked with the attR1 and attR2 sequences was inserted into the *US2* site in the MDV genome using the Counter Selection BAC Modification Kit (Gene Bridges, Berkeley, CA, USA) according to the manufacturer’s instructions. To insert the CAGW-*env* and CAGW-*gag*-IRES-*env* cassettes into the *US2* site, the modified fosmid 103-US2-KanR-ccdB (Figure 1C) was mixed with either the pENTR-CAGW*-env* or the pENTR-CAGW-*gag-*IRES-*env* plasmid and treated with LR Clonase II Enzyme (Invitrogen). The mixtures were then transformed into competent *Escherichia coli* EPI300-T1 cells (Epicentre, Madison, WI, USA). We then selected for fosmids where the KanR-ccdB sequence had been replaced by the CAG-env or CAG-gag-IRES-env cassette. The resulting fosmids were designated 103-us2-env and 103-us2-gag-IRES-env (Figure 1D).

### 2.3. Recombinant MDV Rescue

Five fosmid combinations with or without foreign insertions that covered the entire MDV genome were used for viral rescue. Viral DNA inserts were released from purified fosmids by digestion with the NotI enzyme (Thermo Fisher Scientific, Franklin, MA, USA) and purified by phenol-chloroform extraction and ethanol precipitation. For reconstitution of viruses, primary CEFs were transfected with purified fosmid DNAs using calcium phosphate [20]. Four days after transfection, cells were trypsinized, seeded into a 100-mm dish, and monitored for cytopathic effects (CPEs). CPE-positive samples were harvested for further characterization of recombinant MDVs (rMDVs), designated as rMDV/ALV-env and rMDV/ALV-gag+env.

### 2.4. Confirmation of Env and Gag Expression

Expression of Env and Gag from the rMDVs was confirmed using the immunofluorescence assay (IFA) and Western blotting. For IFA, CEFs in 6-well tissue culture plates were infected with 100 plaque-forming units (PFU) of the rescued viruses. The primary antibodies used were gp85-specific mouse monoclonal antibody (Env) and p27-specific mouse monoclonal antibody (Gag). The secondary antibodies were fluorescein-labeled goat anti-mouse immunoglobulin G (IgG) antibody (Sigma, St. Louis, MO, USA). Cells were observed with a fluorescence microscope (Life, Carlsbad, CA, USA). Western blotting was performed as described previously [7], with gp85-specific mouse monoclonal antibody, p27-specific mouse monoclonal antibody [21], and mouse monoclonal anti-actin antibody (Sigma) as primary antibodies. IRDye 800CW goat anti-mouse IgG (H + L) (LiCor BioSciences, Bad Homburg, Germany) was used as the secondary antibody.

### 2.5. Stability and Growth Properties of rMDVs

To evaluate the genetic stability of the foreign genes in the rMDVs, we passaged the viruses in CEFs 20 times. Detection of the inserted gene was performed by PCR with primers specific for rMDV/ALV-gag+env (5′-GCCACCATGGAAGCCGTCATAAAGGTGA and 5′-TGGGTGTGCCCATAATCGCCA) and rMDV/ALV-env (5′-ATGGAAGCCGTCATAAAGGCA and 5′-TGGGTGTGCCCATAATCGCCA). DNA from passages 5, 10, 15, and 20 were extracted and used as templates for amplification of the inserted gene. Env expression in the 20th passage was detected using IFA, as described above. To investigate the growth properties of the rMDVs, one-step growth analyses were performed, as described previously [22].

### 2.6. Protective Efficacy of rMDV Vaccination

In total, 45 one-day-old SPF chickens were randomly divided into three groups, with 15 chickens in each group. Two groups were inoculated subcutaneously on the back of the neck with 5000 PFU rMDV/ALV-env or rMDV/ALV-gag+env. The third group was mock-injected with phosphate-buffered saline (PBS). Serum was collected weekly after vaccination until the chickens were challenged. The chickens from each inoculated group (except for five no-challenge control chickens in the mock-injected group) were challenged intraperitoneally with 1000 50% Tissue Culture Infective Dose (TCID_50_) of ALV-J JL093-1 at four weeks of age, and the chickens were monitored daily for signs of illness. The body, spleen, and bursa of the Fabricius of each chicken was weighed 45 days post-challenge. The development indices of the spleen and bursa of Fabricius were calculated using the following equation: (organ weight)/(body weight) × 1000.

This study was carried out in strict accordance with the recommendations in the Guide for the Care and Use of Laboratory Animals of the Ministry of Science and Technology of the People’s Republic of China. The protocol was approved by the Committee on the Ethics of Animal Experiments of the Harbin Veterinary Research Institute, Chinese Academy of Agricultural Sciences (approval number JQ-YA-02).

### 2.7. Serologic Tests and Viremia Detection

Blood samples were aseptically collected from all chickens in heparinized tubes before and after challenge infection at weekly intervals. To determine the anti-ALV-J antibody titers in the sera, a commercial ALV-J antibody enzyme-linked immunosorbent assay (ELISA) test kit (IDEXX, Beijing, China) was used in accordance with the manufacturer’s protocol. For ALV viremia detection, DF1 cells were inoculated with plasma samples from the chickens and then incubated at 37 °C and 5% CO_2_ for 7 days. The cells were checked for the presence of the virus using ALV p27 antigen ELISA test kits (IDEXX).

### 2.8. Statistical Analysis

The intergroup differences were analyzed using GraphPad software (version 6.0 for Windows, GraphPad Software, La Jolla, CA, USA) followed by *t*-tests of multiple comparisons. *p* < 0.05 was considered to represent statistically significant differences.

## 3. Results

### 3.1. Rescue of rMDVs

**Figure 2:**
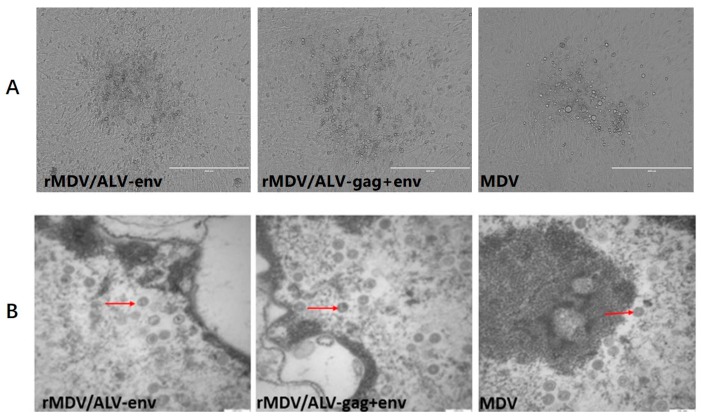
Characterization of recombinant Marek’s disease herpesviruses (rMDVs) with the avian leucosis virus subgroup J (ALV-J) *gag* and *env* gene insertions. (**A**) the cytopathic effects (CPE) induced by the recombinant MDVs containing the gag and env genes and the parental virus (MDV) in chicken embryo fibroblasts (CEFs); (**B**) transmission electron microscopy of rMDV and parent MDV. **Red** arrows indicate MDV particles. Scale bars = 200 nm.

The *US2* region in the MDV genome has been identified as being nonessential for viral replication and suitable for foreign gene insertion [23,24,25,26]. We therefore constructed two cassettes, one containing the *env* gene and one containing the *gag*-IRES-*env* gene of ALV-J strain JL093-1, and inserted each of them into the *US2* region of the MDV genome. To rescue recombinant MDVs, the modified fosmids containing *env* and *gag*-IRES-*env* were used to replace the parental fosmid 103 for transfection. After passaging once in CEFs, MDV1-typical plaques appeared in the CEFs transfected with the DNA combinations (Figure 2A). Typical MDV particles (Figure 2B) resulting from the recombination of overlapping fosmid DNA fragments were observed by transmission electron microscopy 12–13 days after transfection, indicating that viruses containing the *env* or *gag*-IRES-*env* genes within the MDV US2 region were successfully generated.

### 3.2. Expression of Env and Gag

**Figure 3:**
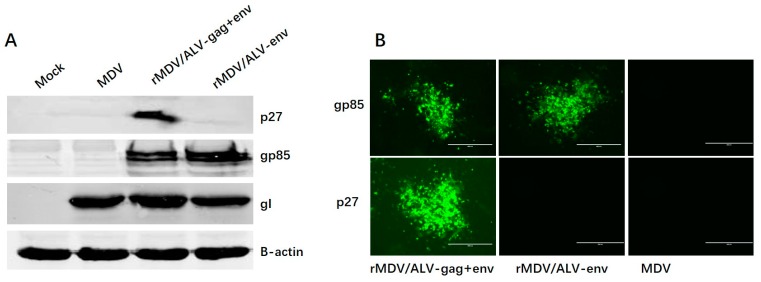
Western blot and immunofluorescence analyses of the recombinant Env and Gag proteins expressed in the rMDV-infected CEFs. The primary antibodies used were gp85-specific mouse monoclonal antibody (Env) and p27-specific mouse monoclonal antibody (Gag). (**A**) Western blotting of recombinant MDVs expressing Gag and Env proteins from ALV-J. Chicken β-actin and MDV gI protein in the cell lysates was used as the internal control; and (**B**) detection of Env and Gag protein expression in the recombinant virus-infected CEFs using immunofluorescence.

Expression of the ALV-J Env and Gag proteins from rMDVs was assessed by Western blot and IFA. Western blot analyses were performed using lysates of cells infected with rMDV/ALV-env or rMDV/ALV-gag+env. The constituent proteins were probed using gp85-specific (Env) and p27-specific (Gag) mouse monoclonal antibodies (Figure 3A). The levels of Env protein expressed by the two recombinant viruses differed, with Env expression being slightly higher in rMDV/ALV-env than that in rMDV/ALV-gag+env. Env and Gag protein expression was also confirmed by indirect IFA (Figure 3B). As expected, cells infected with the parent MDV did not react with either antibody. Both cells infected with rMDV/ALV-env and rMDV/ALV-gag+env, however, reacted with the gp85-specific mouse monoclonal antibody, and rMDV/ALV-gag+env also reacted with the p27-specific mouse monoclonal antibody. These results confirm that both rMDVs express the Env antigen, and rMDV/ALV-gag+env also expresses the Gag protein.

Gag virus-like particles (VLPs) self-assemble when the human immunodeficiency virus (HIV) *gag* gene is expressed in a cell line. Similarly, when HIV *gag* and *env* genes are expressed together, Gag-Env VLPs are created [27]. To explore whether the co-expression of the *gag* and *env* genes produced ALV-J pseudovirions, we sought the presence of ALV-J Gag-Env pseudovirions in cells infected with rMDV/ALV-gag+env by immunoelectron microscopy. However, ALV-J Gag-Env pseudovirions were not observed. This indicates that ALV-J pseudovirions are not created following co-expression of the *gag* and *env* genes in rMDV/ALV-gag+env.

### 3.3. In Vitro Replication and Stability of rMDVs

**Figure 4:**
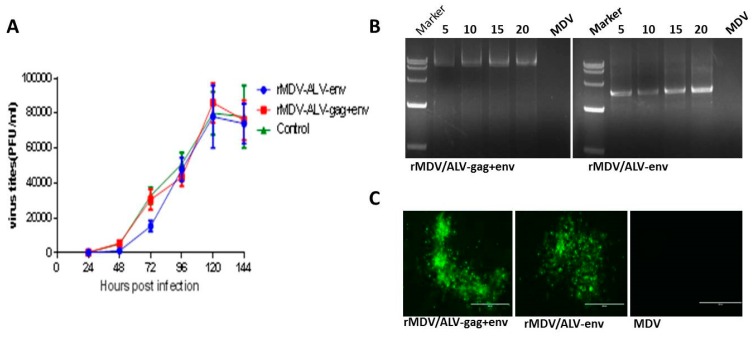
Growth kinetics and genetic stability of rMDV/ALV-gag+env and rMDV/ALV-env (**A**) growth curves (one-step growth kinetics) of rMDV/ALV-gag+env, rMDV/ALV-env, and parent MDV; (**B**) detection of the insertion of the *gag* and *env* genes in the recombinant viruses after passaging. Numbers indicate the passages of the recombinant viruses. The size marker is DL15000; and (**C**) detection of Env protein expression in recombinant virus-infected CEFs following 20 passages using immunofluorescence.

To test whether the *env* or *gag*-IRES-*env* insertions affected the in vitro replication of MDV, the growth curves (Figure 4A) of the two rMDVs were compared with that of the parent MDV. The rMDVs replicated to levels similar to that of the parent MDV, indicating that the insertion of the *env* and *gag* genes did not affect the replication of the MDV vaccine strain.

The two rMDVs were sequentially passaged 20 times in CEFs to determine the genetic stability of the virus. Following this, the *env* and *gag* genes could both be detected in the corresponding recombinants by PCR amplification (Figure 4B). The results revealed no variability in the size of the *env* or *gag* gene products following repeated passaging, indicating that the genes were stably inserted into the MDV genome. Similarly, no differences in the expressions of the recombinant Env and Gag proteins were observed following IFA analysis of rMDV-infected cells after every fifth passage (Figure 4C).

### 3.4. Antibody Responses against ALV-J Induced by rMDVs in Chickens

**Figure 5:**
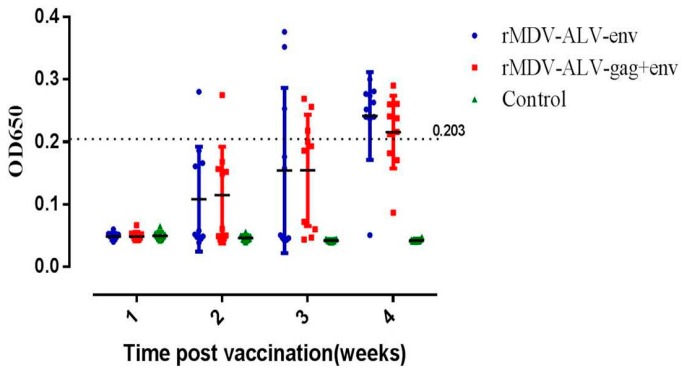
Antibody response against ALV-J virus in chickens inoculated with the recombinant Marek’s disease herpesviruses (MDVs). Serum antibody expression was determined 1–4 weeks post-inoculation using an IDEXX ELISA test kit. Serum samples with an OD650 value higher than a critical value were considered ALV-J-antibody positive. This critical value was determined by the following equation: [(mean of sample OD650) − (mean of negative control OD650)] × 0.6/[(mean of positive control OD650) − (mean of negative control OD650)]. The dashed line indicates the positive critical value.

Groups of one-day-old chicks immunized with rMDV/ALV-env or rMDV/ALV-gag+env produced ALV-J gp85-specific antibodies, as measured by ELISA of serum samples collected 1–4 weeks post-vaccination. The earliest detection of an immune response to the gp85 glycoprotein was two weeks post-vaccination (Figure 5). The ratio of antibody-positive chickens was 8/10 in the rMDV/ALV-env group and 7/10 in the rMDV/ALV-gag+env group at four weeks post-vaccination. These data indicate that an MDV vector vaccine expressing ALV-J antigens is able to elicit serum antibody responses against ALV-J in vivo.

### 3.5. Vaccine Efficacy against ALV-J Challenge in Chickens

**Figure 6:**
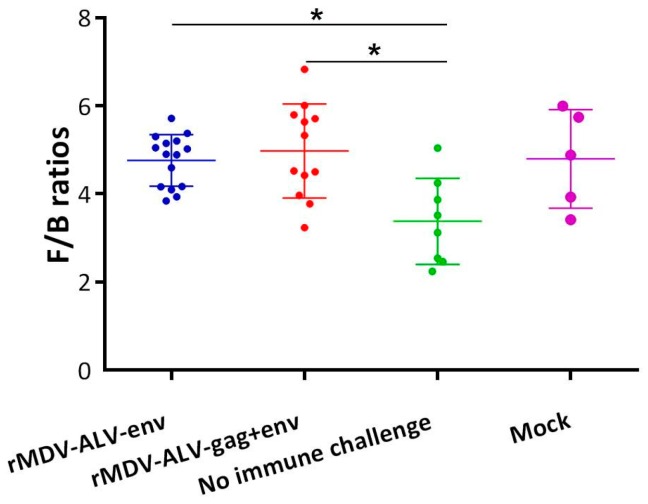
Vaccination with recombinant Marek’s disease herpesviruses (MDVs) protects against immunosuppressive lesions in specific-pathogen-free (SPF) chickens 35 days after challenge with avian leucosis virus subgroup J (ALV-J). Statistical analysis was performed using the paired *t*-test. * *p* < 0.05. F/B ratio = bursa weight/body weight × 1000.

To determine the efficacy of rMDV vaccination against ALV-J, the vaccinated and control groups (excluding the no-challenge group) were challenged with ALV-J strain JL093-1. The protective efficacy of the rMDV vaccines was evaluated based on the viremia rates of challenged chickens. The non-vaccinated challenged control group exhibited a viremia rate of 55.5% seven days post-challenge, while the viremia rate dropped to 26.7% and 33.3% for chickens inoculated with rMDV/ALV-env or rMDV/ALV-gag+env, respectively (Table 1). After 14 days, 25% of the chickens in the challenged control group were still positive for viremia; in contrast, no chickens in either of the rMDV-vaccinated groups were positive. Furthermore, the development indices of the bursa of Fabricius, which were examined at the end of the animal experiment (Figure 6), showed that the bursae in the vaccinated group were comparable with those of the non-challenged control group, while the challenged control group exhibited notable bursal atrophy. These results suggest that both rMDVs provided effective protection against ALV-J challenge in chickens.

## 4. Discussion

MDV and ALV-J are major immunosuppressive viruses that cause significant economic losses to the chicken industry in China. Co-infection of MDV and ALV-J frequently occurs in chicken flocks, causing greater losses than a single infection [28]. Marek’s disease can be successfully controlled by vaccination with attenuated or non-pathogenic MDV strains [29]. However, there is currently no effective vaccine for the prevention of ALV-J infection. Therefore, the main goal of this study was to evaluate MDV as a vector for the delivery of a vaccine capable of protecting chickens against ALV-J. We manipulated a fosmid clone carrying the MDV genome to incorporate expression cassettes for the *env* and *gag* genes from ALV-J. The resulting fully infectious rMDVs exhibited stable expression of recombinant Env and Gag proteins. Administration of a single dose of live rMDV/ALV-env or rMDV/ALV-gag+env to one-day-old chicks conferred immunity against pathogenic ALV-J challenges. These results indicate that both rMDVs have the potential to prevent ALV-J infection. This is the first study describing the potency and protective efficacy of an rMDV against ALV-J challenge.

The classical methods for constructing recombinant MDVs, such as plasmid transfection and viral DNA co-transfection, can be time consuming and labor intensive due to the need for plaque purification. In addition, a selection marker must remain in the genome [30]. Bacterial artificial chromosomes (BACs) are powerful tools for constructing recombinant MDVs [31]. However, the presence of the BAC vector sequence in the viral genome often causes genetic and phenotypic alterations, and the excision of the BAC sequence is difficult [32,33,34]. Here, we utilized a high-efficiency and reliable fosmid system for rescuing recombinant MDVs that does not require a selection marker. Furthermore, the fosmid sequence is removed by restriction endonuclease NotI before transfection. With this system, we generated an rMDV within two weeks, demonstrating that the use of overlapping cosmid DNAs to generate recombinant MDVs may be a convenient and efficient method for MDV genetic manipulation and MDV-based vector vaccine construction.

There are several factors affecting the efficacy of recombinant MDV serotype 1 vaccines [23]. A strong promoter that results in higher protein expression provides better protective efficacy [35]. In previous studies, the CMV and SV40 promoters have generally been used to drive expression of foreign genes in rMDVs [22,23,36]. In this study, the *env* and *gag*-IRES-*env* genes were expressed under control of the hybrid CMV enhancer/chicken β-actin promoter, which has been reported to be more efficient than the conventional CMV promoter [37]. In addition, a WPRE was inserted downstream of the *env* and *gag*-IRES-*env* genes. This element was previously reported to enhance antigen expression from DNA vaccines and retroviral vectors [19,38]. The MDV insertion site of the foreign gene also affects the immunogenicity and vaccine efficacy of recombinant MDVs [23]. In this study, we used the *US2* insertion site. The *US2* sites of HVT [39] and MDV1 [40] have been used as insertion sites for foreign genes in development of recombinant HVTs or MDVs. In our preliminary studies, we inserted the green fluorescent protein gene (GFP) into several MDV insertion sites to examine the effect of insertion site on the expression levels of foreign genes (unpublished data). Our results suggested that the expression level of GFP was particularly high when the gene was inserted into the *US2* site. Therefore, for the development of effective rMDV vaccines, the US2 region likely provides the optimal site for gene insertion. MDV, as a herpesvirus, infects chickens persistently; therefore, vaccine immunity may also continue to the end of the chickens’ lives. MDV vaccines as a recombinant vaccine vector can induce the expression of foreign antigens and induce antibody production continuously. Therefore, we speculate that our rMDV vaccines may induce breeder hens to produce effective maternal antibodies to protect the hatched chickens against early ALV-J infection.

VLPs are self-assembling bionanoparticles that expose multiple epitopes on their surface and faithfully mimic native virions. VLPs can enhance the immune response owing to the multiplicity of arrayed virion-like epitopes on the VLP surface [41]. HIV-1 pseudovirions are capable of exhibiting the native Env trimer on their membrane surfaces [42]. The use of an uncleaved Gag core and a primary isolate Env should result in the production of pseudovirions that can present Env for induction of a strong humoral immune response [27,43]. The goal of constructing rMDV/ALV-gag+env in the present study, therefore, was to explore whether the co-expression of the gag and env genes produces ALV-J pseudovirions. However, ALV-J pseudovirions were not generated in cells infected with rMDV/ALV-gag+env. This may be because the levels of Gag and Env expression were not high enough for the formation of VLPs. Furthermore, as expression of the IRES-dependent second gene is less efficient than that of the first gene under both in vitro and in vivo conditions [18], the expression of Env in rMDV/ALV-gag+env was slightly lower than that in rMDV/ALV-env. Consequently, the protective efficacy against ALV-J challenge provided by rMDV/ALV-gag+env inoculation in chickens was also slightly weaker than that provided by rMDV/ALV-env. In addition, in China, antigen tests are often used for the purification of ALV-infected flocks by ELISA, but antibody tests are rarely used [44,45]. Thus, rMDV-gag+env may affect differential diagnosis in practical applications, while rMDV-env will not. Therefore, we will only consider rMDV/ALV-env as a candidate vaccine strain for future research.

There is a significant correlation between protective efficacy and levels of antibody production in inoculated chickens [27,46]. Serological data revealed that antibodies against ALV-J gradually increased in SPF chickens vaccinated with rMDV/ALV-env or rMDV/ALV-gag+env, with detectable levels of antibody production 14 days after vaccine administration. By 28 days after administration, most chickens had generated ALV-J antibodies, which is why the chickens were challenged with ALV-J at 4 weeks of age. Following challenge with ALV-J, most chickens vaccinated with either rMDV/ALV-env or rMDV/ALV-gag+env were negative for viremia, while most chickens that did not receive either vaccine were positive for infection. Another problem is that the resistance of ALV infection is related with the age of chickens [47]. Although the challenged control group only had 55% viremia, the protection in the vaccinated and control groups showed a significant difference. Therefore, it can be concluded that these MDV vector vaccines for ALV-J should effectively reduce the rate of positive viremia in chicken flocks. In addition, the research and development of our rMDV vaccines are in the initial stage. The main goal of this study was to confirm the potential of these rMDV vaccines to prevent ALV-J. We will confirm the immunity effect of our rMDV vaccines in commercial animals in future studies.

Since its introduction in China in the 1980s, MDV-1 814 has served as an important live vaccine for the prevention of highly virulent MDV infection. The vaccine has been used widely in China and has been effective in limiting the spread of MDV infection [48,49]. The effectiveness and safety of this vaccine has been fully confirmed. Furthermore, previous work has indicated that the efficacy of MDV1 vaccines is associated with the replication of the MDV vaccine strain [29]. The insertion of the *env* and *gag* genes at the US2 site did not affect the replication of the MDV vaccine strain. The *US2* sites of MDV1 have been used as insertion sites for foreign genes in the development of recombinant MDVs in many previous studies, which did not affect the protective efficacy of the recombinant MDVs against challenges with a highly virulent MDV strain [40]. In addition, in another study conducted by our laboratory, we inserted the *VP2* gene of IBDV into the US2 site. The ability of the rMDV-VP2 to protect chickens from MDV challenge was as good as that of the parent MDV1 vaccine strain of the recombinant virus. Consequently, we believe that it is not necessary to add the evaluation of the protective efficacy of recombinant MDVs against challenges from a highly virulent MDV strain.

In summary, we successfully constructed the first recombinant MDV vaccine expressing ALV-J antigenic genes that provides a protective effect against ALV-J. This confirms the potential of rMDV vaccines for the clinical prevention of ALV-J. In addition, because of the incidence of MDV and ALV-J co-infection, our study suggests the future possibility of a multivalent MDV vaccine capable of protecting chickens against both ALV-J and MDV.

## Figures and Tables

**Table 1:** ALV-J infection ratios and protection ratios in the chickens from 1 to 3 weeks after being challenged with ALV-J.

Group	1st Week		2nd Week	
	P.V.R	P.R.	P.V.R.	P.R.
rMDV/ALV-env	26.7% (4/15)	73.3% (11/15)	0.0% (0/15)	100.0% (15/15)
rMDV/ALV-gag+env	33.3% (4/12)	67.7% (8/12)	0.0% (0/12)	100% (12/12)
No immune challenge	55.5% (5/9)	45.5% (4/9)	25.0% (2/8)	75% (6/8)
No immune no challenge	0.0% (0/5)	100% (5/5)	0.0% (0/5)	100% (5/5)

Note. P.V.R.: positive viremia ratios; P.R.: protection ratios.

## References

[1] Nakamura K., Ogiso M., Tsukamoto K., Hamazaki N., Hihara H., Yuasa N. (2000). Lesions of bone and bone marrow in myeloid leukosis occurring naturally in adult broiler breeders. Avian Dis..

[2] Xu B., Dong W., Yu C., He Z., Lv Y., Sun Y., Feng X., Li N., Lee L.F., Li M. (2004). Occurrence of avian leukosis virus subgroup J in commercial layer flocks in China. Avian Pathol..

[3] Payne L.N., Brown S.R., Bumstead N., Howes K., Frazier J.A., Thouless M.E. (1991). A novel subgroup of exogenous avian leukosis virus in chickens. J. Gen. Virol..

[4] Gao Y., Yun B., Qin L., Pan W., Qu Y., Liu Z., Wang Y., Qi X., Gao H., Wang X. (2012). Molecular epidemiology of avian leukosis virus subgroup J in layer flocks in China. J. Clin. Microbiol..

[5] Sun S., Cui Z. (2007). Epidemiological and pathological studies of subgroup J avian leukosis virus infections in Chinese local “yellow” chickens. Avian Pathol..

[6] Cheng Z.Q., Zhang L., Liu S.D., Zhang L.J., Cui Z.Z. (2005). Emerging of avian leukosis virus subgroup J in a flock of Chinese local breed. Wei Sheng Wu Xue Bao.

[7] Zhang L., Cai D., Zhao X., Cheng Z., Guo H., Qi C., Liu J., Xu R., Zhao P., Cui Z. (2014). Liposomes containing recombinant gp85 protein vaccine against ALV-J in chickens. Vaccine.

[8] Dou W., Li H., Cheng Z., Zhao P., Liu J., Cui Z., Liu H., Jing W., Guo H. (2013). Maternal antibody induced by recombinant gp85 protein vaccine adjuvanted with CpG-ODN protects against ALV-J early infection in chickens. Vaccine.

[9] Xu Q., Ma X., Wang F., Li H., Xiao Y., Zhao X. (2015). Design and construction of a chimeric multi-epitope gene as an epitope-vaccine strategy against ALV-J. Protein Expr. Purif..

[10] Osterrieder N., Kamil J.P., Schumacher D., Tischer B.K., Trapp S. (2006). Marek’s disease virus: From miasma to model. Nat. Rev. Microbiol..

[11] Calnek B.W. (2001). Pathogenesis of Marek’s disease virus infection. Curr. Top. Microbiol. Immunol..

[12] De Boer G.F., Groenendal J.E., Boerrigter H.M., Kok G.L., Pol J.M. (1986). Protective efficacy of Marek’s disease virus (MDV) CVI-988 CEF65 clone C against challenge infection with three very virulent MDV strains. Avian Dis..

[13] Rispens B.H., van Vloten H., Mastenbroek N., Maas H.J., Schat K.A. (1972). Control of Marek’s disease in the Netherlands. I. Isolation of an avirulent Marek’s disease virus (strain CVI 988) and its use in laboratory vaccination trials. Avian Dis..

[14] Luschow D., Werner O., Mettenleiter T.C., Fuchs W. (2001). Protection of chickens from lethal avian influenza A virus infection by live-virus vaccination with infectious laryngotracheitis virus recombinants expressing the hemagglutinin (H5) gene. Vaccine.

[15] Sakaguchi M., Nakamura H., Sonoda K., Okamura H., Yokogawa K., Matsuo K., Hira K. (1998). Protection of chickens with or without maternal antibodies against both Marek’s and Newcastle diseases by one-time vaccination with recombinant vaccine of Marek’s disease virus type 1. Vaccine.

[16] Sonoda K., Sakaguchi M.H., Yokogawa K., Tokunaga E., Tokiyoshi S., Kawaguchi Y., Hirai K. (2000). Development of an Effective Polyvalent Vaccine against both Marek’s and Newcastle Diseases Based on Recombinant Marek’s Disease Virus Type 1 in Commercial Chickens with Maternal Antibodies. J. Virol..

[17] Zhang F., Liu C.J., Zhang Y.P., Li Z.J., Liu A.L., Yan F.H., Cong F., Cheng Y. (2012). Comparative full-length sequence analysis of Marek’s disease virus vaccine strain 814. Arch. Virol..

[18] Mizuguchi H., Xu Z., Ishii-Watabe A., Uchida E., Hayakawa T. (2000). IRES-dependent second gene expression is significantly lower than cap-dependent first gene expression in a bicistronic vector. Mol. Ther. J. Am. Soc. Gene Ther..

[19] Li K., Li G., Gao H., Qi X., Gao Y., Qin L., Wang Y., Wang X. (2013). Protection of chickens against reticuloendotheliosis virus infection by DNA vaccination. Vet. Microbiol..

[20] Morgan R.W., Cantello J.L., McDermott C.H. (1990). Transfection of chicken embryo fibroblasts with Marek’s disease virus DNA. Avian Dis..

[21] Delong L.I., Liu Z., Yun B., Guan W.U., Gao Y., Qin L., Xiaole Q.I., Wang Y., Gao H., Liu S. (2012). Expression of Capsid Protein p27 Gene of Avian Leucosis Virus and Preparation of Multiclonal Antibodies against Recombinant p27 Protein. China Poult..

[22] Schumacher D., Tischer B.K., Fuchs W., Osterrieder N. (2000). Reconstitution of Marek’s disease virus serotype 1 (MDV-1) from DNA cloned as a bacterial artificial chromosome and characterization of a glycoprotein B-negative MDV-1 mutant. J. Virol..

[23] Cui H., Gao H., Cui X., Zhao Y., Shi X., Li Q., Yan S., Gao M., Wang M., Liu C. (2013). Avirulent Marek’s disease virus type 1 strain 814 vectored vaccine expressing avian influenza (AI) virus H5 haemagglutinin induced better protection than turkey herpesvirus vectored AI vaccine. PLoS ONE.

[24] Jarosinski K.W., Margulis N.G., Kamil J.P., Spatz S.J., Nair V.K., Osterrieder N. (2007). Horizontal transmission of Marek’s disease virus requires US2, the UL13 protein kinase, and gC. J. Virol..

[25] Jang H.K., Ono M., Kim T.J., Izumiya Y., Damiani A.M., Matsumura T., Niikura M., Kai C., Mikami T. (1998). The genetic organization and transcriptional analysis of the short unique region in the genome of nononcogenic Marek’s disease virus serotype 2. Virus Res..

[26] Cantello J.L., Anderson A.S., Francesconi A., Morgan R.W. (1991). Isolation of a Marek’s disease virus (MDV) recombinant containing the lacZ gene of Escherichia coli stably inserted within the MDV US2 gene. J. Virol..

[27] Hicar M.D., Chen X., Briney B., Hammonds J., Wang J.J., Kalams S., Spearman P.W., Crowe J.E. (2010). Pseudovirion particles bearing native HIV envelope trimers facilitate a novel method for generating human neutralizing monoclonal antibodies against HIV. J. Acquir. Immune Defic. Syndr..

[28] Witter R.L. (1997). Avian tumor viruses: Persistent and evolving pathogens. Acta Vet. Hung..

[29] Baigent S.J., Smith L.P., Nair V.K., Currie R.J. (2006). Vaccinal control of Marek’s disease: Current challenges, and future strategies to maximize protection. Vet. Immunol. Immunopathol..

[30] Cunningham C., Davison A.J. (1993). A cosmid-based system for constructing mutants of herpes simplex virus type 1. Virology.

[31] Brune W., Messerle M., Koszinowski U.H. (2000). Forward with BACs: New tools for herpesvirus genomics. Trends Genet..

[32] Zhao Y., Petherbridge L., Smith L.P., Baigent S., Nair V. (2008). Self-excision of the BAC sequences from the recombinant Marek’s disease virus genome increases replication and pathogenicity. Virol. J..

[33] Wagner M. (1999). Systematic excision of vector sequences from the BAC-cloned herpesvirus genome during virus reconstitution. J. Virol..

[34] Zhou F., Li Q., Wong S.W., Gao S.J. (2010). Autoexcision of bacterial artificial chromosome facilitated by terminal repeat-mediated homologous recombination: A novel approach for generating traceless genetic mutants of herpesviruses. J. Virol..

[35] Wang S., Taaffe J., Parker C., Solorzano A., Cao H., Garcia-Sastre A., Lu S. (2006). Hemagglutinin (HA) proteins from H1 and H3 serotypes of influenza A viruses require different antigen designs for the induction of optimal protective antibody responses as studied by codon-optimized HA DNA vaccines. J. Virol..

[36] Lee L.F., Lupiani B., Silva R.F., Kung H.J., Reddy S.M. (2008). Recombinant Marek’s disease virus (MDV) lacking the Meq oncogene confers protection against challenge with a very virulent plus strain of MDV. Vaccine.

[37] Jiang Y., Yu K., Zhang H., Zhang P., Li C., Tian G., Li Y., Wang X., Ge J., Bu Z. (2007). Enhanced protective efficacy of H5 subtype avian influenza DNA vaccine with codon optimized HA gene in a pCAGGS plasmid vector. Antivir. Res..

[38] Han J.J., Mhatre A.N., Wareing M., Pettis R., Gao W.Q., Zufferey R.N., Trono D., Lalwani A.K. (1999). Transgene expression in the guinea pig cochlea mediated by a lentivirus-derived gene transfer vector. Hum. Gene Ther..

[39] Baigent S.J., Petherbridge L.J., Smith L.P., Zhao Y., Chesters P.M., Nair V.K. (2006). Herpesvirus of turkey reconstituted from bacterial artificial chromosome clones induces protection against Marek’s disease. J. Gen. Virol..

[40] Tsukamoto K., Kojima C., Komori Y., Tanimura N., Mase M., Yamaguchi S. (1999). Protection of Chickens against Very Virulent Infectious Bursal Disease Virus (IBDV) and Marek’s Disease Virus (MDV) with a Recombinant MDV Expressing IBDV VP2. Virology.

[41] Zhao Q., Li S., Yu H., Xia N., Modis Y. (2013). Virus-like particle-based human vaccines: Quality assessment based on structural and functional properties. Trends Biotechnol..

[42] Crooks E.T., Moore P.L., Franti M., Cayanan C.S., Zhu P., Jiang P., Vries R.P.D., Wiley C., Zharkikh I., Schülke N. (2007). A comparative immunogenicity study of HIV-1 virus-like particles bearing various forms of envelope proteins, particles bearing no envelope and soluble monomeric gp120. Virology.

[43] Berkower I., Raymond M., Muller J., Spadaccini A., Aberdeen A. (2004). Assembly, structure, and antigenic properties of virus-like particles rich in HIV-1 envelope gp120. Virology.

[44] Zhang M.J., Zhang S., Xiao-Xue G.U., Yuan L., Wang B.Y., Zhai X.Y., Tian K.G. (2012). Epidemiological Study of Avian Leukemia in Different Import Poultry Strains from 2009 to 2010 in Some Areas of China. China Anim. Husb. Vet. Med..

[45] Pavón J., Galvis O., Echeverría F., Castaño J.G., Echeverry M., Robledo S., Jiménez-Piqué E., Mestra A., Anglada M. (2013). Development of an antigen-capture ELISA for the detection of avian leukosis virus p27 antigen. J. Virol. Methods.

[46] Li Y., Reddy K., Reid S.M., Cox W.J., Brown I.H., Britton P., Nair V., Iqbal M. (2011). Recombinant herpesvirus of turkeys as a vector-based vaccine against highly pathogenic H7N1 avian influenza and Marek’s disease. Vaccine.

[47] Fadly A.M., Smith E.J. (1999). Isolation and some characteristics of a subgroup J-like avian leukosis virus associated with myeloid leukosis in meat-type chickens in the United States. Avian Dis..

[48] Tong K. (1984). Study on the Immunization of Chickens against Marek’s Disease—Report on a Naturally Avirulent Vaccine Strain of Marek’ Disease Herpesvirus. Chin. J. Anim. Vet. Sci..

[49] Zhang F., Liu C.J., Zhang Y.P., Liu A.L., Yan F.H., Hou G.Y., Cong F. (2010). Sequencing analysis of repeat regions of Marek’s disease virus vaccine strain 814. Chin. J. Prev. Vet. Med..

